# Letter from S. T. Overstreet, M. D., Live Oak, Fla.

**Published:** 1891-12

**Authors:** S. T. Overstreet

**Affiliations:** Live Oak, Fla.


LIVE OAK, FLA., November 25th, 1891.

*Editors Atlanta Medical and Surgical Journal, Atlanta, Ga.:*

DEAR SIR—We have a little girl in this county four years old who is a fully developed woman in every respect, and was that way at two years and nine months. Can this be excelled in any land or country? Very respectfully,

S. T. OVERSTREET, M. D.

---

Since our last appearance, three of Atlanta's prominent physicians have entered the holy bonds and delightful estate of wedlock—Dr. J. W. Hood, Dr. C. C. Greene and Dr. Clarence Johnson. The gentlemen and their better halves will please accept the JOURNAL's distinguished considerations and best wishes.

---

WE regret to announce that Dr. Kennedy, of the JOURNAL, is undergoing an attack of typhoid fever. At this writing the disease seems to be under control, and we presume Dr. Kennedy will soon be convalescing.

---

At any given time in the United States it is estimated that there are two million persons afflicted with syphilis.

